# *tableone*: An open source Python package for producing summary statistics for research papers

**DOI:** 10.1093/jamiaopen/ooy012

**Published:** 2018-05-23

**Authors:** Tom J Pollard, Alistair E W Johnson, Jesse D Raffa, Roger G Mark

**Affiliations:** Massachusetts Institute of Technology (MIT), MIT Laboratory for Computational Physiology, Cambridge, Massachusetts, USA

**Keywords:** descriptive statistics, python, quantitative research

## Abstract

**Objectives:**

In quantitative research, understanding basic parameters of the study population is key for interpretation of the results. As a result, it is typical for the first table (“Table 1”) of a research paper to include summary statistics for the study data. Our objectives are 2-fold. First, we seek to provide a simple, reproducible method for providing summary statistics for research papers in the Python programming language. Second, we seek to use the package to improve the quality of summary statistics reported in research papers.

**Materials and Methods:**

The *tableone* package is developed following good practice guidelines for scientific computing and all code is made available under a permissive MIT License. A testing framework runs on a continuous integration server, helping to maintain code stability. Issues are tracked openly and public contributions are encouraged.

**Results:**

The *tableone* software package automatically compiles summary statistics into publishable formats such as CSV, HTML, and LaTeX. An executable Jupyter Notebook demonstrates application of the package to a subset of data from the MIMIC-III database. Tests such as Tukey’s rule for outlier detection and Hartigan’s Dip Test for modality are computed to highlight potential issues in summarizing the data.

**Discussion and Conclusion:**

We present open source software for researchers to facilitate carrying out reproducible studies in Python, an increasingly popular language in scientific research. The toolkit is intended to mature over time with community feedback and input. Development of a common tool for summarizing data may help to promote good practice when used as a supplement to existing guidelines and recommendations. We encourage use of tableone alongside other methods of descriptive statistics and, in particular, visualization to ensure appropriate data handling. We also suggest seeking guidance from a statistician when using *tableone* for a research study, especially prior to submitting the study for publication.

## OBJECTIVES

Research is highly dependent on the quality of its underpinning data. To assist with the interpretation of an analysis, biomedical research guidelines typically include recommendations for describing the data with summary statistics. The CONSORT (CONsolidated Standards of Reporting Trials) guidelines, for example, indicate the importance of a “table showing baseline demographic and clinical characteristics for each group”. The authors note that this information “allows readers, especially clinicians, to judge how relevant the results of a trial might be to an individual patient”.^1^ Other popular reporting guidelines, such as those found on the EQUATOR (Enhancing the QUAlity and Transparency Of health Research) Network, offer similar advice.^2^

It is typical for the first table of a biomedical research paper, the so called “Table 1”, to provide the baseline characteristics of the patient population. The presentation of this table is relatively consistent across studies, showing statistics such as number and proportions of patients, means and medians, and the frequency of missing data. The measures may be stratified across a categorical variable such as the study’s primary outcome in order to show how the population characteristics differ between subgroups. While the computation of summary statistics is conceptually straightforward, the technical task is typically cumbersome and offers ample opportunities for the introduction of misleading and avoidable errors through flaws in data entry, coding mistakes, and incorrect table formatting.

**Table 1.** Example of a table produced by the tableone package when applied to a small subset of data from MIMIC-III

Variables	Level	Is null	Overall
*n*			1000
Age (years), median (IQR)		0	68 (53–79)
SysABP (mmHg), mean (SD)		291	114.25 (40.16)
Height (cm), mean (SD)		475	170.09 (22.06)
Weight (pounds), mean (SD)		302	82.93 (23.83)
ICU type, *n* (%)	CCU	0	162 (16.2)
	CSRU		202 (20.2)
	MICU		380 (38.0)
	SICU		256 (25.6)
In-hospital mortality, *n* (%)	0	0	864 (86.4)
	1		136 (13.6)

Warnings about inappropriate summaries of the data are raised during generation and displayed below the table.

Warning, Hartigans Dip Test reports possible multimodal distributions for: Age, Height, SysABP.

Warning, Tukey rule indicates far outliers in: Height.

IQR: interquartile range; SysABP: systolic arterial blood pressure; ICU: intensive care unit.

A recently published Correction in JAMA Psychiatry, titled “Errors in Table 1”, offers an example: “the rate of 300.096 was replaced with 30.0096; and for a maternal age of older than 40 years, the rate of 73.199 was replaced with 7.3199”.^3^ Another recent correction in the New England Journal of Medicine notes that “‘Nonelective’ should have been ‘Elective’” in the summary of the clinical trial population.^4^ These kind of errors are easy to make, difficult to detect, and happen in many studies, not just the examples provided here.

Providing software to simplify the creation of Table 1 has several benefits: reduction in time spent tediously calculating and formatting results, prevention of common errors when creating summary statistics, and greater consistency in reporting summary statistics. Yoshida and Bohn^5^ created a package in the programming language R to automatically create the relevant summary statistics in the appropriate format. This package has become increasingly popular among researchers using R. To date, there is no analogous software to produce a similar table in Python.

We sought to provide a simple, reproducible method for creating summary statistics for research papers in the Python programming language, which has become increasingly popular for scientific studies in recent years. In addition, we sought to encourage better practice for study reporting by highlighting issues relating to the appropriateness of summary statistics. The package is maintained as a public project named *tableone*, enabling the research community to develop a centralized toolkit that can help to promote reproducible, better quality reporting of data characteristics as they mature over time. These technical tools are intended to complement recommendation documents and guidelines for reporting on research studies.

## BACKGROUND AND SIGNIFICANCE

The Statistical Analyses and Methods in the Published Literature (SAMPL) Guidelines note that reporting errors are common in published biomedical literature.^6^ Citing several studies, the authors suggest that the problem of poor statistical reporting is “long-standing, widespread, [and] potentially serious” and that this problem is common even in “the world’s leading peer-reviewed general medical and specialty journals”. While we might expect statistical errors to arise mostly in more complex areas of analysis, it appears that the problem concerns mostly basic statistics. A commentary on how to detect and prevent errors in medical literature suggests that virtually all of the errors in question deal with misuse of material discussed in most introductory statistics textbooks.^7^

As an example, a commonly reported issue is the use of standard error of the mean, rather than standard deviation, as a summary of data variability. The suggestion is that this occurs either due to tradition or, more worryingly, as a result of researcher bias because “the standard error of the mean is always smaller than the standard deviation”.^7^ In an editorial titled *Ten Rules for Reading Clinical Research Reports*, Yancey insists the reader should “Question the validity of all descriptive statistics”, echoing this common and inappropriate use of standard error of the mean.^8^

The extent to which a biomedical journal can and should review the methodology of submitted papers is an open question for editors. In Statistical Reviewing Policies of Medical Journals, the author explains that a large barrier to methodologic reviews is the availability of resources for doing so.^9^ Where a statistical reviewer does happen to be available, it is still common for data and code to be unavailable, and our own experiences have shown that simply reproducing the patient cohort of a study is non-trivial at best.^10^^,^^11^ According to Glantz, many statisticians would prefer not to spend their time “grinding out garden-variety statistics for other people”, and that the job of summarizing data is often best done by the investigators themselves.^7^ This is not to give the job of a statistician to a clinical researcher, but to allow the researcher to carry out introductory statistics, while leaving the more complex statistical tasks and reviews to the expert statisticians.

## MATERIALS AND METHODS

Python is a rapidly growing programming language with a number of mature libraries for data analysis.^12^ Researchers are increasingly using Python due to its large and active scientific computing community, ease of interactive data analysis, and utility as a general purpose programming language.^13^ The software library Pandas is central to conducting data analysis in Python.^14^ Pandas introduces a DataFrame object which simplifies manipulation of structured datasets. When working with a DataFrame, Pandas provides a number of convenient routines to calculate averages, medians, and other aggregate measures. *tableone* utilizes DataFrames to summarize and present data, leveraging the popularity of Pandas among the scientific community and the excellent integration of Pandas with literate computing approaches such as Jupyter Notebooks.^15^^,^^16^

Our aim in developing *tableone* is to provide a simple, reproducible method for providing summary statistics for research papers in the Python programming language. In doing this, we provide features such as: automatic detection of categorical variables; reporting of *P*-values with adjustments for multiple hypothesis testing; grouping of measures by a variable such as the primary outcome; and customizable formatting options. Variables defined as normally distributed are summarized by mean and standard deviation by default, while non-normally distributed variables are summarized by median and interquartile range.

Mean and standard deviation are often poor estimates of the center or dispersion of a variable’s distribution when the distribution: is asymmetric, has “fat” tails and/or outliers, contains only a very small finite set of values or is multimodal. Median and interquartile range may offer a more robust summary than mean and standard deviation for skewed distributions or in the presence of outliers, but may be misleading in cases such as multimodality. Several tests have therefore been incorporated to raise potential issues with reported summary statistics. For example, Hartigan’s Dip Test is computed and a warning message is generated if the test results indicate a possible multimodal distribution.^7^^,^^17^ Similarly, Tukey’s Rule highlights outliers in distributions that may distort the mean. While formal statistical checks can be useful in detecting potential issues, they often are not very useful in small sample sizes so these tests should be used alongside standard visualization methods.^18^

When multiple hypotheses are tested, as may be the case when numerous variables are summarized in a table, there is a higher chance of observing a rare event. To help address this issue, corrections for multiple comparisons have been implemented.^19^ By default, the package computes the Bonferroni correction, which addresses the issue in a simple way by dividing the prespecified significance level (Type I error rate, α) by the number of hypothesis tests conducted. This approach is known to over-correct, effectively reducing the statistical power of the tests, particularly when the number of hypotheses are large or when the tests are positively correlated. There are many alternatives which may be more suitable and also widely used, and which should be considered in situations that would be adversely affected by the conservative nature of the Bonferroni correction.^20–22^

The *tableone* package was developed following good practice guidelines for scientific computing.^23^ The code is openly available on GitHub under a permissive MIT License, enabling continuous, collaborative development.^24^ Issues are tracked publicly in the repository and guidelines for contributing to the package are provided, promoting transparency and helping to ensure that the software functionality meets the demand of the scientific community. Contributions that address known issues such as feature developments and bug fixes are actively encouraged. A continuous integration server is used to test new contributions, adding an additional level of quality control to proposed changes. Package dependencies, defined in the repository, include Pandas, NumPy, SciPy, and StatsModels.^25–28^

## RESULTS

The *tableone* package has been published on the Python Package Index (PyPI), a repository of software for the Python programming language. It is therefore straightforward to install using the standard installation command: “pip install tableone”. The dataset to be summarized must be provided as a Pandas DataFrame, structured so that each row captures a unique case (eg a patient) and each column pertains to an observation associated with the case (eg patient age or a laboratory test result).

After importing the package into the Python environment, the simplest application of it is to create an instance of the TableOne class with the DataFrame to be summarized (“data”) as a single input argument, as follows:

mytable = TableOne(data)

In this case, the package will create a new DataFrame containing the summary statistics, automatically identifying continuous and categorical variables within the data and summarizing them appropriately. Once generated, the table may be viewed on screen or exported to a range of established formats, including LaTeX, CSV, and HTML using the “*to_format()*” methods (for example, “mytable.to_latex()”). When the table is generated, automated tests will print a series of remarks that highlight potential issues to the researcher. For example, if outliers are indicated by Tukey’s rule, the researcher is warned to consider the implications of this with respect to the summary statistics.

**Figure 1.**
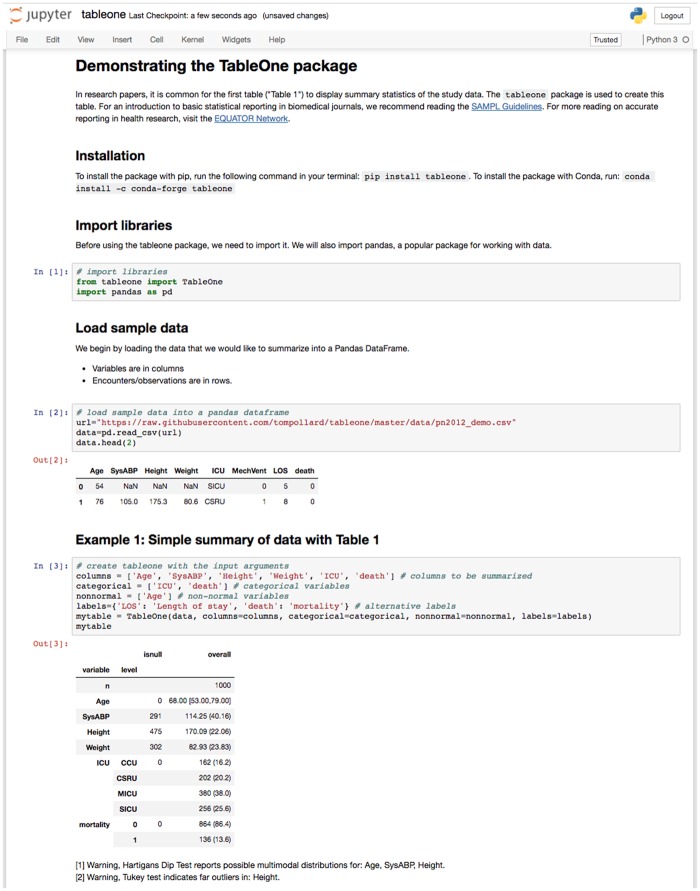
A executable Jupyter Notebook provides worked examples for applying the TableOne package to exemplar data.

**Figure 2.**
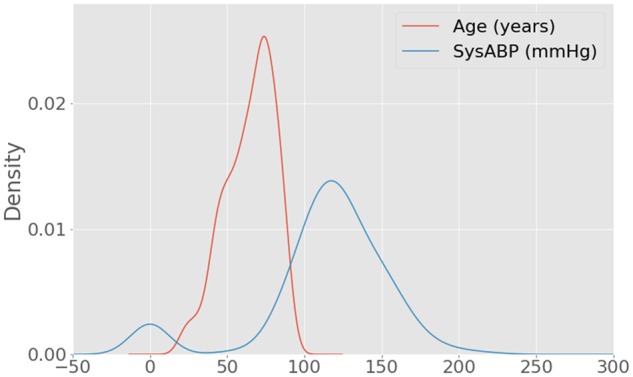
A test for modality raises a warning message for both “Age” and “SysABP” (systolic arterial blood pressure). Upon inspection, SysABP shows clear peaks at both ∼0 and ∼120.

**Figure 3.**
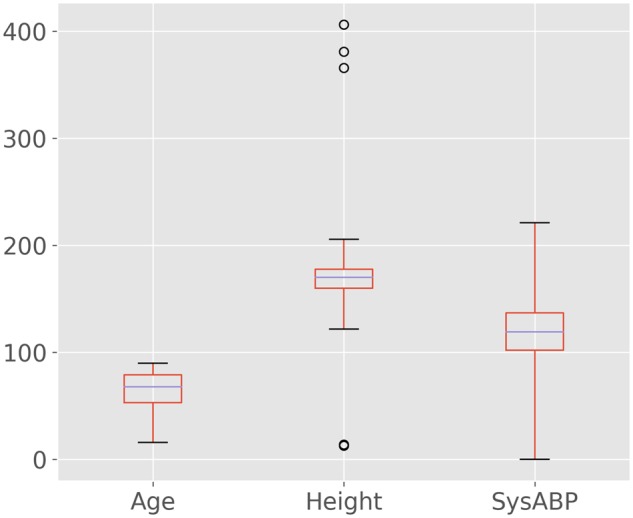
Box-plot of 3 variables with whiskers located at a distance of three times the interquartile range. Points outside these whiskers are labeled “far outliers” and denoted by circles. A test for far outliers with Tukey’s rule raises a warning for height but not age or systolic arterial blood pressure (SysABP).

We provide an executable Jupyter Notebook alongside the code that demonstrates the application of the package to a small cohort of patients in MIMIC-III (Figure 1). MIMIC-III is a large, publicly available dataset of critically ill patients admitted to intensive care units (ICUs) at the Beth Israel Deaconess Medical Center in Boston, MA, USA.^29^ The example subset corresponds to 1000 patients who stayed at least 48 h in the ICU and contains demographics, treatment, and survival status at hospital discharge. Table 1 shows an example of the output of the tableone package, and Table 2 shows the first 5 rows of the dataset prior to summarization. Figure 2 shows a kernel smoothed density for the Age and SysABP variables, highlighting the multimodality concerns raised by the *tableone* package. Figure 3 shows a box-plot of the data, with circles indicating outlying points warned about by Tukey’s test. The package is under continuous development, so for up-to-date information we suggest reviewing the package documentation, which is available online.^30^

**Table 2.** Example of the data used, showing the first 5 rows

Age	SysABP	Height	Weight	ICU	MechVent	LOS	death
54	NaN	NaN	NaN	SICU	0	5	0
76	105.0	175.3	80.6	CSRU	1	8	0
44	148.0	NaN	56.7	MICU	0	19	0
68	NaN	180.3	84.6	MICU	0	9	0
88	NaN	NaN	NaN	MICU	0	4	0

Each row captures a unique case (eg a patient) and each column pertains to an observation associated with the case (eg patient age).

NaN: Not a Number; SysABP: systolic arterial blood pressure; ICU: intensive care unit; SICU: surgical ICU; CSRU: cardiac surgery recovery unit; MICU: medical ICU; MechVent: mechanical ventilation; LOS: hospital length of stay.

## DISCUSSION

We encourage use of *tableone* alongside other methods of descriptive statistics and, in particular, visualization to ensure appropriate data handling. When used in this way, the package helps researchers to create summary statistics for study populations, an integral task for almost any research study. The default settings have been carefully chosen to match the preferences of most researchers and to adhere to best practices, with the intention that only minor configurations are generally necessary when generating the table. Such configurations would include specifying grouping variables (such as study outcome), adding alternative labels for variable names, and limiting the number of levels to display for a categorical variable.

In providing a reproducible approach to generating a summary table from a dataset, we hope to reduce the contribution of coding and data entry errors to misreported statistics. The consistency of a standardised approach will help to discourage some of the common reporting issues discussed previously. Automated tests for issues such as multimodality and outliers will raise warnings for the researcher, helping to catch and prevent potentially misleading summary statistics before they are reported. Plotting the distribution of each variable by group level via histograms, kernel density estimates and boxplots is a crucial component to data analysis pipelines, however, and these tests are not intended to replace such methods. Visualization is often is the only way to detect problematic variables in many real-life scenarios.

By default we do not support statistical hypothesis tests for comparison of distributions, because as a general rule we believe that it is best practice not to do so.^1^^,^^2^^,^^6^^,^^31^ However, as has been highlighted elsewhere, many journals still require *P*-values alongside summary statistics.^32^ In their guidelines for authors, for example, the New England Journal of Medicine include the following statement: “For tables comparing treatment groups at baseline in a randomized trial (usually the first table in the manuscript), significant differences between or among groups (ie, *P* < 0.05) should be identified in a table footnote and the *P*-value should be provided in the format specified above.”^33^ To encourage the wider adoption of methods which account for multiple comparisons, we have implemented methods such as the Bonferroni and Sidak corrections.

Sharing a tool such as *tableone* creates a responsibility to promote better practice and to avoid propagating poor practice, and we are committed to working with the research community to ensure this is done. Documentation and example code will be continuously improved and used to encourage authors to observe study reporting guidelines. Statistical referees of research studies using *tableone* should benefit from the fact that their feedback can be fed into the package for future users, helping to promote good practice within a community rather simply being directed at the authors of a single study. In addition, referees carrying out detailed methodological code reviews on a study-by-study basis should find it more straightforward to assess a single function call to *tableone* (with publicly discussed strengths and weaknesses) than to review custom code for this task in each case.

## CONCLUSION

We describe the release of the *tableone* package for Python. The package provides a reproducible approach for compiling summary statistics for research papers into a publishable format. The package will be continuously improved and updated, based on community feedback, and encourage good practices for scientific reporting. It should be noted that while we have tried to follow best practices, automation of even basic statistical tasks can be unsound if done without supervision. We, therefore, suggest seeking guidance from a statistician when using *tableone* for a research study, especially prior to submitting the study for publication.

## FUNDING

The authors were supported by grants NIH-R01-EB017205 and NIH-R01-EB001659 from the National Institutes of Health.

*Conflict of interest statement*. None declared.

## CONTRIBUTORS

TJP, AEWJ, and JDR developed the software. TJP, AEWJ, JDR, and RGM contributed to the paper and approved the final submission.
